# Puerarin inhibits HDAC1-induced oxidative stress disorder by activating JNK pathway and alleviates acrolein-induced atherosclerosis

**DOI:** 10.1016/j.clinsp.2024.100413

**Published:** 2024-07-18

**Authors:** YeTing Li, XiaoNing Li, Man Zheng, FanLi Bu, ChunYan Xiang, FengLei Zhang

**Affiliations:** Department of Cardiology, Dongying People's Hospital (Dongying Hospital of Shandong Provincial Hospital Group), Dongying City, Shandong Province, China

**Keywords:** Atherosclerosis, Puerarin, Acrolein, Antioxidant Enzymes, HDAC1

## Abstract

**Objective:**

Atherosclerosis (AS) is a common pathogenesis of cardiovascular diseases. Puerarin (Pue) is a Chinese herbal remedy used to prevent and treat AS. Here, this research investigated the effect of Pue on AS progression.

**Methods:**

ApoE^−/−^ mice were induced with acrolein. Body weight, blood lipid index, inflammatory factors, mitochondrial oxidative stress, and lipid deposition were detected. IL-6 and TNF-α were detected by ELISA. Oil red staining and H&E staining were used to observe the aortic sinus plaque lesions. Serum expressions of inflammatory factors IL-6, TNF-a, SOD, GSH and MDA were detected by ELISA, the mRNA expression levels of HDAC1 in the aorta were detected by RT-qPCR, and IL-6 and TNF-α in the aorta were detected by immunohistochemistry. JNK, p-JNK, OPA-1, and HDAC1 were detected by Western blotting.

**Results:**

Pue administration can effectively reduce lipid accumulation in AS mice induced by acrolein. Pue promoted the activity of SOD, GSH and MDA, and inhibited the formation of atherosclerotic plaques and the process of aortic histological changes. Pue reduced IL-6 and TNF-α. HDAC1 expression was down-regulated and p-JNK-1 and JNK protein expression was up-regulated.

**Conclusion:**

Pue reduces inflammation and alleviates AS induced by acrolein by mediating the JNK pathway to inhibit HDAC1-mediated oxidative stress disorder.

## Highlights

• Pue inhibits weight gain in atherosclerotic mice.
• Pue inhibits serum lipid levels in atherosclerotic mice.
• Pue inhibits inflammation in atherosclerotic mice.
• Pue inhibits the formation of AS lesions.

## Introduction

Cardiovascular disease is mainly caused by atherosclerosis, a chronic, low-grade inflammatory disease that affects the large and medium arteries.^1^ Many factors accelerate the atherogenic process, such as the release of inflammatory chemokines and cytokines and the production of Reactive Oxygen Secies (ROS).^2^ Innate and adaptive immune responses can promote or inhibit AS, and some signaling pathways associated with the inflammatory response are associated with AS.^3^ Oxidative stress caused by the excessive production of ROS has become a key and ultimate common mechanism of AS. Antioxidants act as checkpoints against ROS, leading to oxidative stress when there is an imbalance between oxidative and antioxidative mechanisms.^4^ Therefore, targeting inflammation and oxidative stress has been considered a treatment direction for AS.^5^^,^^6^

Puerarin (Pue), a natural isoflavone mainly derived from Pueraria lobata (Willd.) Ohwi, has been developed as injection, eye drops, and microemulsion.^7^ Modern pharmacological studies have shown that Pue can effectively improve cardiovascular and cerebrovascular diseases, it has the effects of lowering blood lipids and blood sugar, and it has a positive effect on AS and coronary heart disease, etc.^8^ Pue has a wide range of pharmacological effects and significant protective effects and takes advantage of protecting against organ ischemia-reperfusion injury.^9^ Moreover, Pue has inhibitory effects on the progression of AS such as reducing endothelial damage, anti-inflammation, interfering lipid metabolism, protecting ischemia-reperfusion injury, and anti-myocardial remodeling.^10^ Pue has suggested cardioprotective effects on AS.^11^^,^^12^^,^^8^ It has been noted that Inflammatory factors and oxidative stress injuries may be inhibited by Pue as it can increase mitochondrial antioxidant potential and reduce excessive ROS production. In recent years, it has been reported that the isoflavones in Pue have the effects of dilating blood vessels and coronary arteries, improving microcirculation, anti-arrhythmia, and lowering blood pressure and blood lipids.^13^^,^^14^ Their potential for the prevention and therapy of cardiovascular and cerebrovascular diseases has gradually been discovered and developed, and they are expected to become a new force in the prevention and treatment of AS, but the specific mechanism is still unclear.

This research figured out the mechanism of Pue in AS by JNK signaling to mediate HDAC1-induced oxidative stress disorder, targeting to provide a drug reference for clinical practice in AS.

## Materials and methods

### Animals and reagents

Thirty-two 7-week-old male ApoE^−/−^ mice and eight 20‒22 g wild-type C57BL/6J mice were purchased from SPF(Beijing)BIOTECHNOLOGY Co., Ltd. A specific pathogen-free environment was maintained with temperatures of 20°C and 5 % relative humidity, alternating lighting (12h of illumination and 12h of darkness), and sufficient food and water supply. This experiment was approved by the Animal Ethics Committee of Dongying People's Hospital (Dongying Hospital of Shandong Provincial Hospital Group) (nº 20200375DY). All procedures were carried out complying with the Guiding Principles for the Care and Use of Laboratory Animals. All animal experiments complied with the ARRIVE guidelines. Pue injection (nº H20057087, Tiantai Mountain Pharmaceutical, Chengdu, China).

### Animal model

After 1 week of adapting to the normal diet, the C57BL/6J mice in the control group were fed a normal food diet, while the ApoE^−/−^ mice were randomly divided (n = 8/group). In the sham operation group, model group, and Pue low-, medium-, and high-dose groups, all ApoE^−/−^ mice were fed acrolein (2.5 mg/kg/day) by daily gavage for 8 weeks. Pue low-, medium- and high-dose groups were given 300, 600, 1200 mg.kg^−1^/d^−1^ by gavage until 20 weeks, respectively, and the model group was given an equal dose of normal saline. Eight male C57BL/6J mice of the same age were also given ordinary feed as the control group. After 16 weeks of administration, mice were fasted overnight and anesthetized with isoflurane, and liver weights were recorded. The liver index is equal to the liver wet weight/the body weight of the mouse multiplied by 100 %.

### Assessment of lipids and oxidative stress

Blood was collected from the posterior orbital sinus of the eyeball, and the serum was separated by centrifugation at 4°C at 3500×/g (Sorvall ST-16R, Thermo) for 10 min. Serum TC, TG, LDL-C, and HDL-C levels were measured by enzyme colorimetry. AS Index (AI) = (TC - HDL-C)/HDL-C.

### Oil red O staining

From the proximal root of the aorta to the branch of the iliac artery, cardiac tissue containing the aortic arch was harvested, and the liver tissue was dissected after external fat deposits were removed. The atherosclerotic lesions in the aortic root and lipid accumulation in the liver were analyzed histologically by embedding the heart and liver tissues with OCT compound into 6 μm and 10 μm thick slices, respectively. After staining with oil red O, slice images were collected using a microscope (BA210Digital) and quantified by Image-ProPlus 6.0 software (Media Cybernetics).

### HE-staining

The whole aorta was separated from the root of the aorta to the end of the abdominal aorta. After fixation with 4 % paraformaldehyde, the aorta was kept at 4°C overnight, paraffin-embedded, and continuously sliced into 5 μm to make paraffin sections. Then, HE-staining was conducted, followed by microscopic detection of the aortic AS plaque area.

### Immunohistochemistry

Middle aortic valves were separated, and 5 μm-thick frozen sections were made for immunohistochemical assay, which included the quantification of IL-6 (ab233706, 1: 200; Abcam) and TNF-α (ab1793, 1: 200; Abcam, USA). Results were analyzed using Image Pro Plus 6.0 software.

### ELISA

Serum TNF-α, IL-6, and oxidative stress-related factors (SOD, GSH, and MDA) were detected by ELISA kits.

### JC-1 staining

The mouse aorta was added to JC-1 staining solution (10 mg/mL) and incubated at 37°C for 20 min. Then, the aorta was washed twice with JC-1 dyeing buffer, and fluorescence changes were observed under an inverted microscope.

### RT-qPCR

Total RNA kit extracted total RNA from liver tissue. Total RNA (1 μg) was reverse-transcribed into cDNA. As previously mentioned, gene expression analysis was performed using Fast SYBR Green premix and CFX 96TM real-time System (Bio-Rad). Using the 2^−△△Ct^ method, relative gene expression was calculated by normalizing target genes with β-actin.

### Western blotting

A RIPA buffer containing 1 mM PMSF (Solarbio, Beijing, China) was prepared, and protein from live tissues was harvested by centrifugal detection with the lysis buffer at 12,000 rpm and 4°C for 10 min. The upper layer was obtained to undergo repeated centrifugation and quantified with a BCA protein assay kit (Solarbio). The protein mixed with 5× loading buffer was boiled at 100°C for 10 min, separated on a 10 % SDS-PAGE gel, and transferred to the PVDF membrane at 270 mA for 90 min, and blocked with 5 % skim milk solution at 37°C for 2h. Primary antibodies (1:1000) specific to JNK, p-JNK, OPA-1, and HDAC1 were added to the membrane at 4°C. After TBST rinsing 4 times (10 min each time), the membrane was incubated with a secondary antibody (goat anti-rabbit) at 37°C for 1h and washed with TBST 4 times for 10 min each time. Image-ProPlus 6.0 software was applied for quantitative analysis. Immunoblotting was performed with an ultra-sensitive ECL chemiluminescence kit (Beyotime).

### Statistical analysis

All data were expressed as mean ± Standard Deviation (SD) and processed by the SPSS software program. One-way ANOVA or Bonferroni-adjusted Kruskal-Wallis test was used to present statistical differences; p < 0.05 was considered statistically significant.

## Results

### Pue inhibits weight gain in atherosclerotic mice

**Fig. 1:**
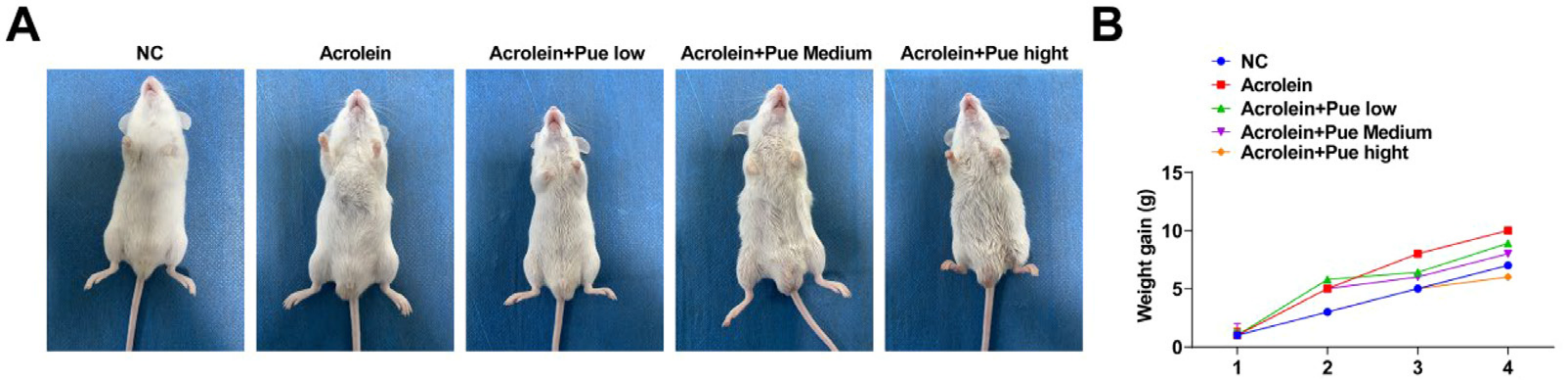
**Pue inhibits weight gain in atherosclerotic mice.** (A) Schematic diagram of the experimental process. (B) Weight gain measurement.

An acrolein-induced ApoE^−/−^ mouse model of AS was established (Fig. 1A). The weight gain trend of acrolein-treated mice was similar to that of the control mice, while the weight gain was inhibited after Pue treatment (Fig. 1B).

### Pue inhibits serum lipid levels in atherosclerotic mice

**Fig. 2:**
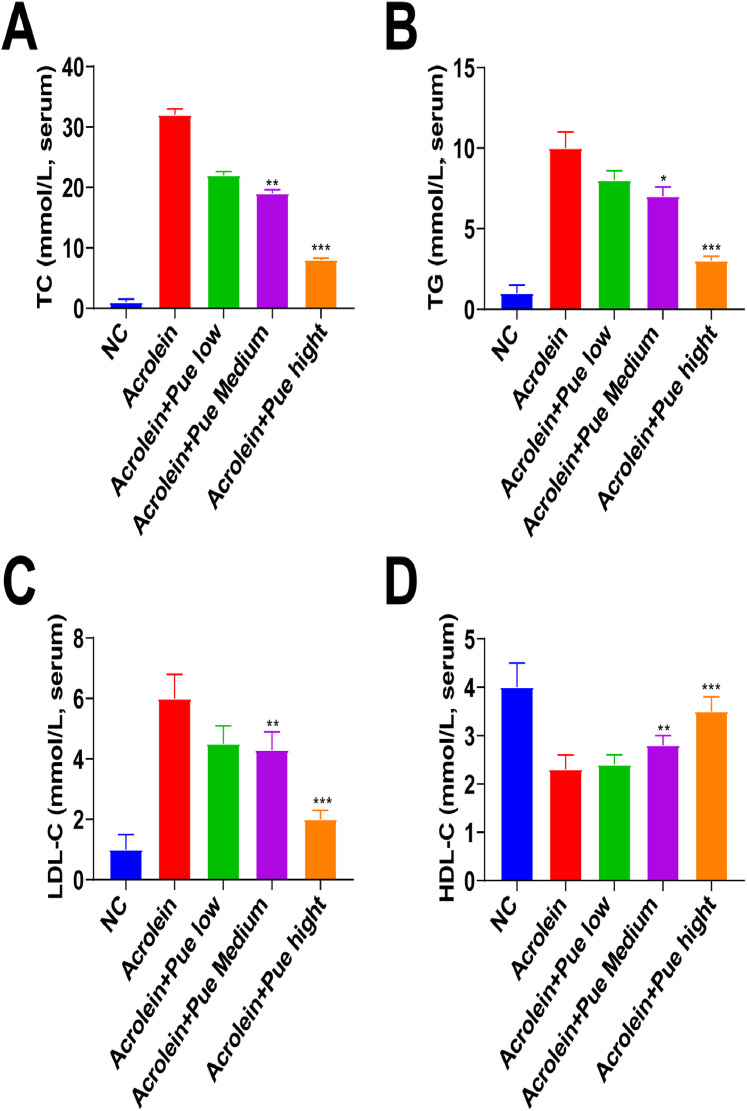
**Pue inhibits serum lipid levels in atherosclerotic mice.** (A‒D) ELISA analysis of serum TG, TC, LDL-C, and HDL-C levels.

Serum TG, TC and LDL-C in atherosclerotic mice were elevated, and HDL-C was reduced (Fig. 2A). Serum TG, TC and LDL-C in each Pue administration group were lowered, and HDL-C was increased (Fig. 2B‒D), with the most significant difference in the high-dose Pue group. The serum lipid level in the high-dose group was lowered mostly by high-dose Pue, and the TG level was particularly decreased.

### Pue inhibits inflammation in atherosclerotic mice

**Fig. 3:**
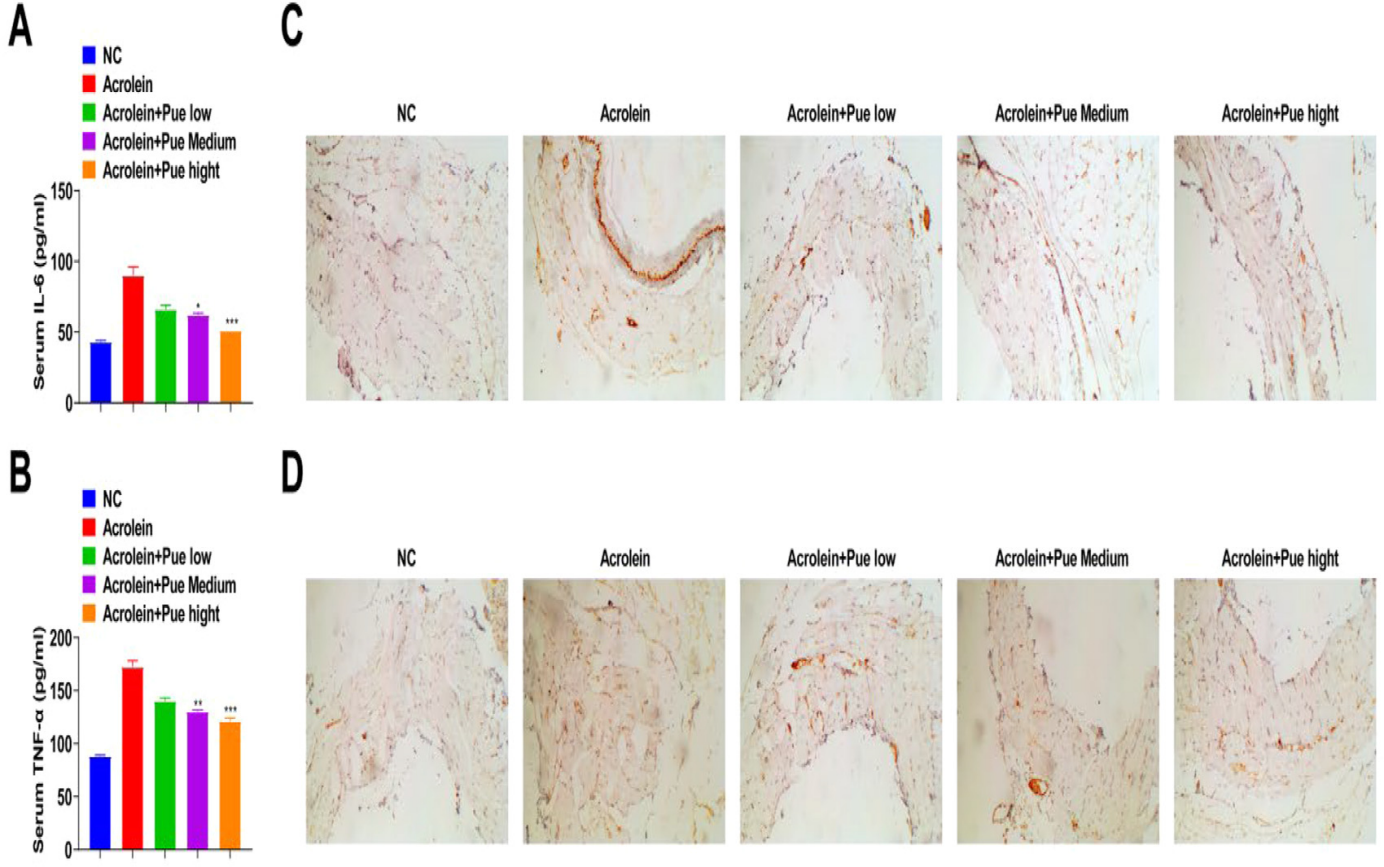
**Pue inhibits inflammation in atherosclerotic mice.** (A‒B) ELISA analysis of serum IL-6 and TNF-α levels. (C‒D) IHC staining of aortic sinus to measure IL-6 and TNF-α.

Serum IL-6 and TNF-α of atherosclerotic mice were enhanced, which were suppressed by Pue administration (Fig. 3A and B). IHC results indicated that the concentrations of IL-6 and TNF-α in plaques in atherosclerotic mice were significantly increased, while reduced after Pue administration (Fig. 3C and D).

### Pue inhibits the formation of AS lesions

**Fig. 4:**
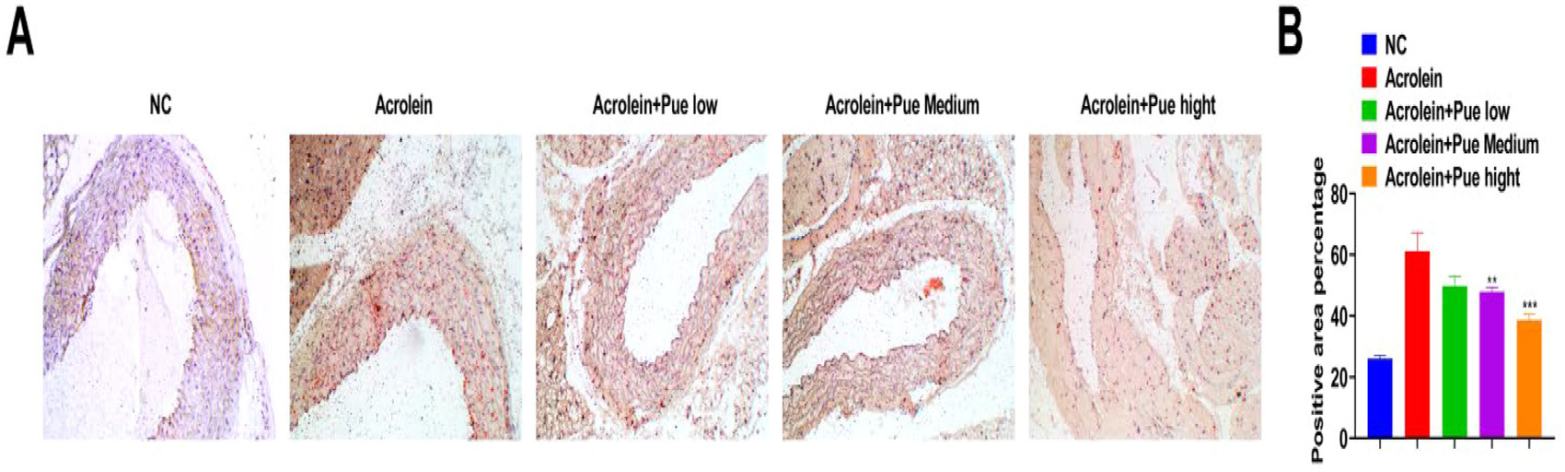
**Pue inhibits the formation of atherosclerotic lesions.** (A) Mouse aortic root oil red O staining. (B) Quantification of the percentage of oil red O positive area.

According to oil red O staining, acrolein induced the formation of atherosclerotic plaques in the aorta of ApoE^−/−^ mice, while there was a varying degree of inhibition of AS lesions in the aortic root following Pue treatment (Fig. 4A). Oil-red O-positive areas in the aortic root was quantified. The aortic plaque in atherosclerotic mice was formed, and the proportion of lipid deposition was 61.26 ± 3.02 %. The aortic root plaque area after Pue treatment was reduced, and the percentage of lipid deposition area was 38.60 ± 6.35 % (Fig. 4B).

### Pue inhibits lipid accumulation in atherosclerotic liver

**Fig. 5:**
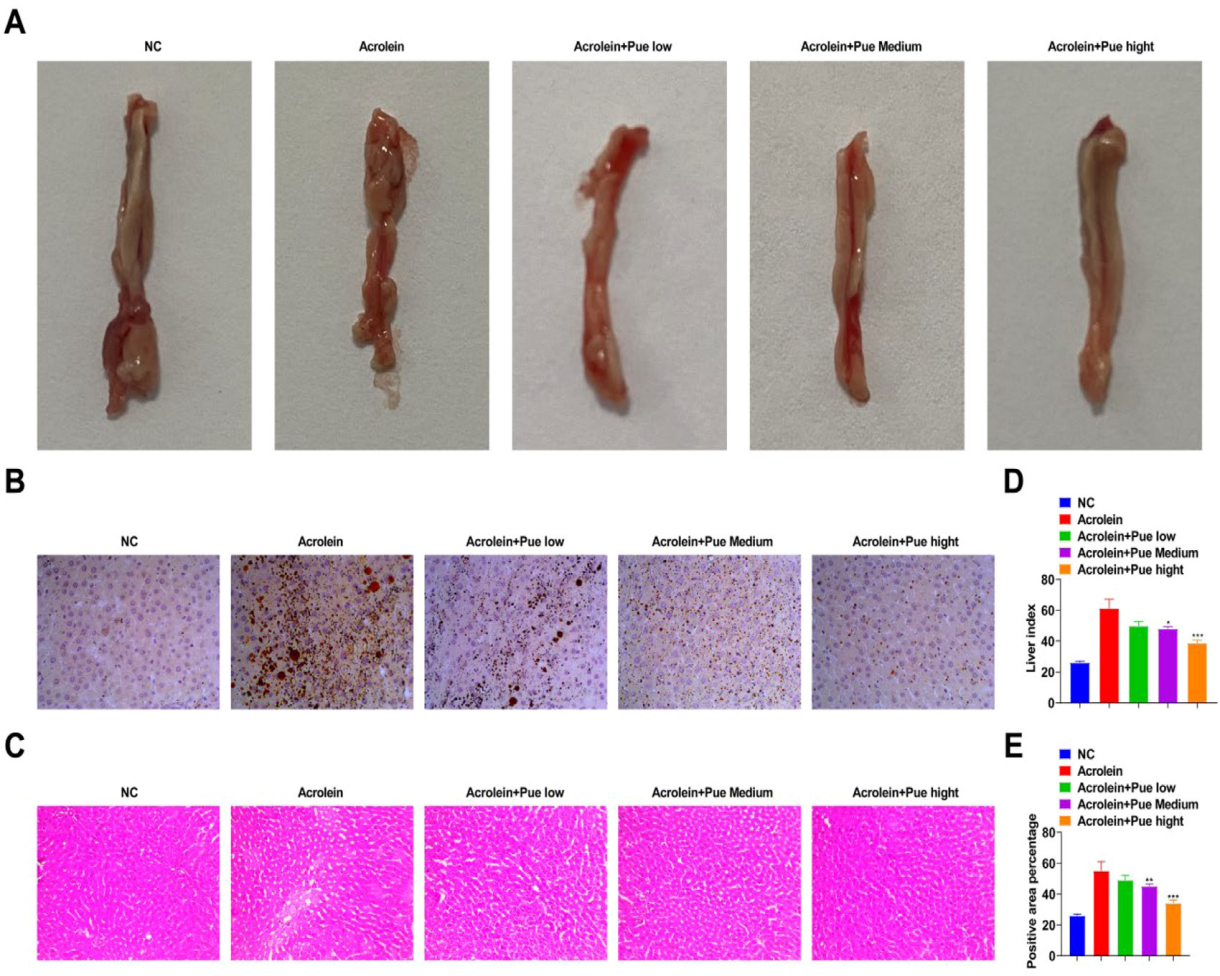
**Pue inhibits lipid accumulation in atherosclerotic liver.** (A) Representative images of livers. (B) Oil Red O-stained liver sections. (C) HE-stained liver sections. (D) Liver index. (E) Percentage quantitative of Oil red O positive area.

Acrolein-fed ApoE^−/−^ mice developed a uniform pale-yellow liver, indicating lipid accumulation in the liver (Fig. 5A). The liver index value of atherosclerotic mice was significantly high. The liver index value induced by acrolein was reversed after Pue treatment (Fig. 5D). Lipid droplet area was quantitatively analyzed by oil red O staining. Lipid droplets were accumulated in the liver of ApoE^−/−^ mice fed with acrolein (Fig. 5B). The area ratio of hepatocyte lipids in the control group and model group was 2.89 ± 1.25 % and 22.01 ± 7.22 %, respectively. Liver lipid accumulation was significantly or extremely significantly decreased after treatment (Fig. 5E). After Pue treatment, the ratio of lipid droplet was 13.04 ± 3.62 %. HE-staining showed that the steatosis of atherosclerotic mice was obvious, the size of lipid droplets was different, hepatocytes were obviously enlarged, and some hepatic sinuses were narrow or even atresia. After treatment, acrolein-induced hepatic steatosis was improved to varying degrees (Fig. 5C). Small and medium steatosis were seen in Pue-treated mice, plus small round vacuoles within the cytoplasm.

### Pue inhibits liver oxidative stress

**Fig. 6:**
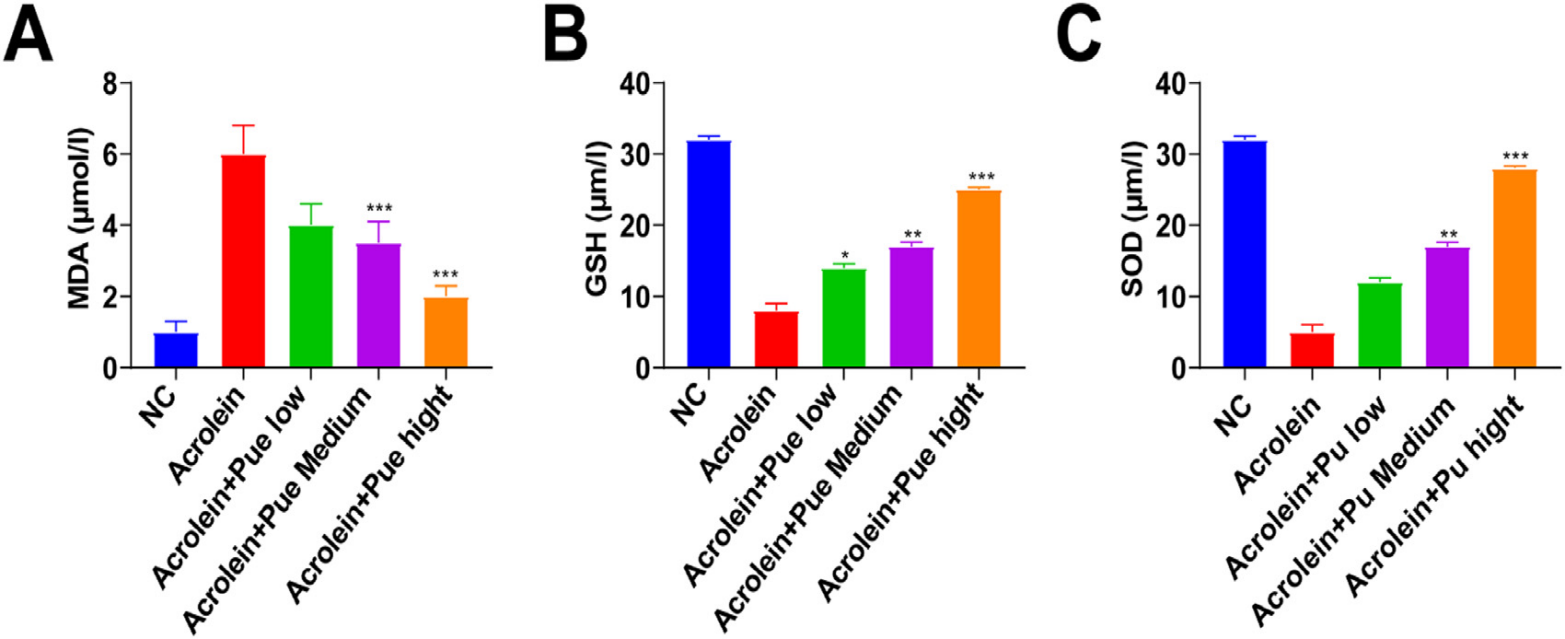
**Liver associated reactive oxidative stress factor levels.** (A‒C) ELISA analysis of SOD, GSH, and MDA in liver.

SOD and GSH levels in atherosclerotic mice were significantly decreased, while MDA levels were increased (Fig. 6A‒C). SOD and GSH levels in all treatment groups were significantly increased and MDA levels were significantly decreased.

### Pue inhibits atherosclerotic mitochondrial membrane potential (MMP)

**Fig. 7:**
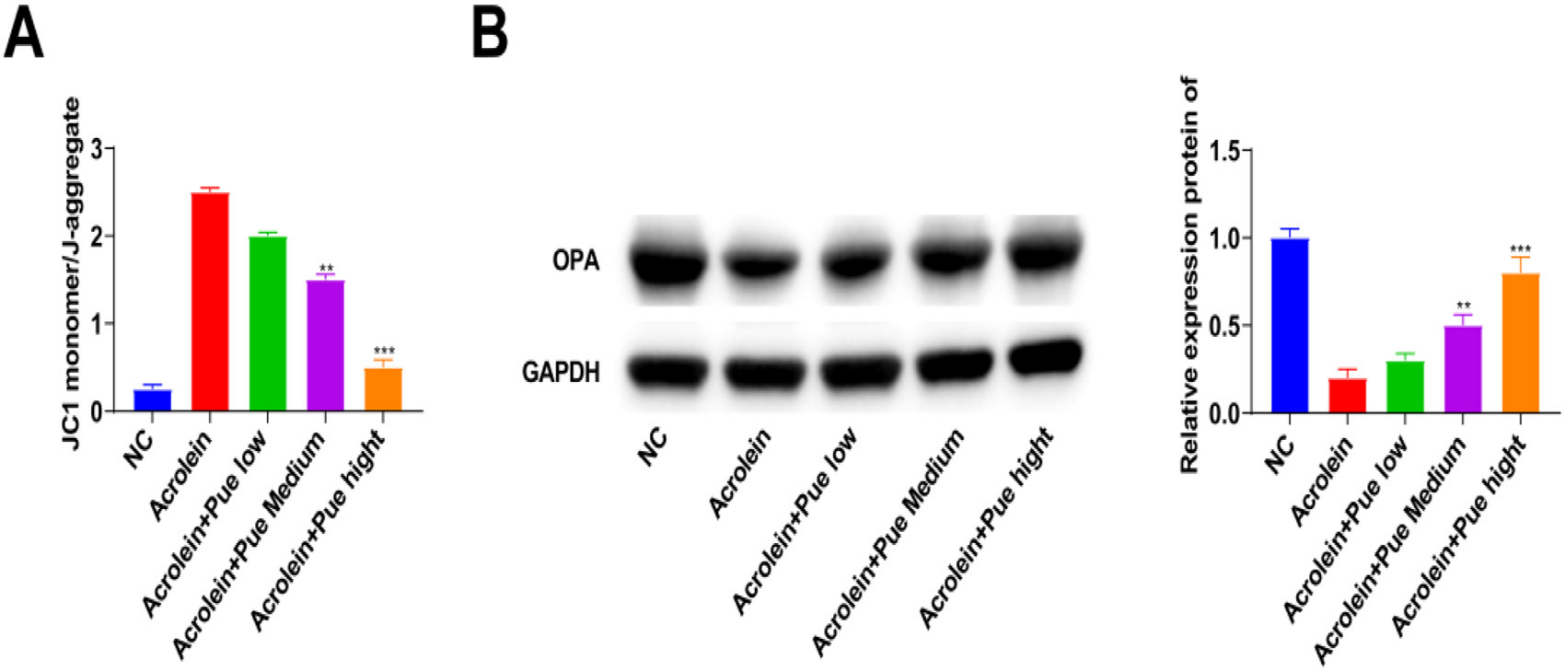
**Pue inhibits atherosclerotic MMP.** (A) JC-1 staining. (B) Western blot measurements of OPA-1.

A change in MMP can be observed by JC-1 staining. The green fluorescence in the aorta of the mice induced by acrolein was enhanced, and the green fluorescence gradually changed to red fluorescence after Pue intervention (Fig. 7A). Mouse aorta mitochondria were damaged by acrolein, resulting in decreased MMP and JC-1 dye uptake. The effect of Pue on mitochondrial function in mouse aortas induced by acrolein was dose-dependent. Mitochondrial oxidative stress-related proteins were detected by Western blotting. With the increase of Pue concentration, OPA-1 expression significantly increased, indicating that Pue has an activation effect on mitochondrial oxidative stress (Fig. 7B).

### Pue activates JNK signaling pathway to inhibit HDAC1 expression

**Fig. 8:**
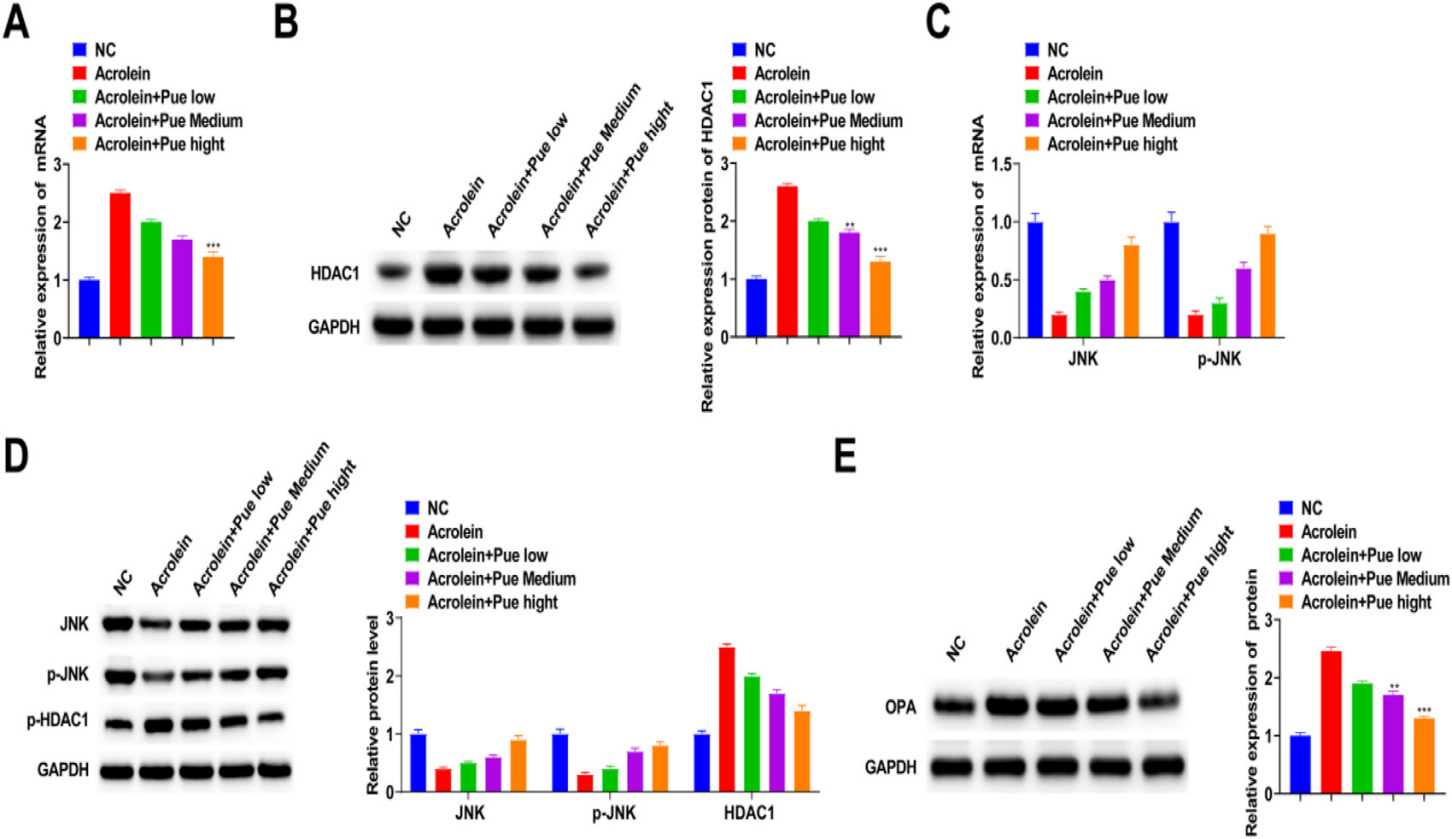
**Pue activates JNK signaling pathway to inhibit HDAC1 expression.** (A) RT-qPCR detection of HDAC1. (B) Western blot measurements of HDAC1. (C) Western blot measurements of phosphorylated HDAC1. (D) Western blot measurements of JNK and p-JNK. (E) Western blot measurements of JNK and p-JNK. (F) Western blot measurements of mitochondria-related proteins.

To investigate whether acrolein-induced oxidative stress is associated with HDAC1, HDAC1 expression was measured by RT-qPCR and Western blotting. HDAC1 levels were reduced after acrolein treatment, but this trend was reversed with the increase of the intervention concentration of Pue (Fig. 8A and B). In addition, Western blotting results showed that phosphorylated HDAC1 expression was downregulated after acrolein treatment, and Pue intervention at different concentrations could significantly reverse its expression, which was related to concentration. This suggests that HDAC1 inhibits mitochondrial oxidative stress in the form of phosphorylation (Fig. 8C).

To further explore whether inhibition of the JNK pathway can improve AS induced by acrolein, related proteins were studied. The results showed that JNK and p-JNK protein expression was elevated in acrolein-induced AS, while the expression was significantly reversed after intervention with a high concentration of Pue, suggesting that Pue could inhibit JNK pathway (Fig. 8D). Next, JNK was activated using Anisomycin. JNK and p-JNK expression was enhanced after activation of the pathway, and the signaling pathway was activated (Fig. 8E). Western blotting results showed that OPA-1 expression was significantly inhibited after activation of this pathway (Fig. 8F). These results further verified that Pue's effect on the oxidative stress activation of atherosclerotic mitochondria induced by acrolein was realized by inhibiting JNK signaling pathway.

## Discussion

Part of the pathology of AS is characterized by chronic inflammation and oxidative stress. Targeting oxidative stress by developing innovative antioxidants or enhancing antioxidant systems is also a proven strategy.^15^ Acrolein is a highly active toxic aldehyde that is a common dietary and environmental contaminant and can also be produced endogenously. Acrolein exposure was positively associated with certain pathological conditions, such as AS. At the cellular level, acrolein induces various harmful effects, especially protein cohesion and oxidative damage.^16^ In the AS mouse model induced by acrolein, the effect of Pue was explored.

Body weight gain is common in atherosclerotic models.^17^ In this research, acrolein increased the body weight of mice, and body weight gain was suppressed after Pue gavage, suggesting the protective effect of Que on AS. Maintaining optimal lipid levels has been considered significant to achieve optimal cardiovascular health.^18^

In addition, dyslipidemia was observed in mice after exposure to acrolein, and Que treatment reduced TC, TG, and LDL-C, while elevating HDL-C in a dose-dependently way, which was consistent with a previous report on AS.^19^ In addition to that, in the field of Type II diabetes mellitus, Pue shows lipid-lowering activity by reducing TC, TG, and LDL-C and improving HDL-C.^20^ At present, it is generally believed that Pue suppresses oxidative stress and inflammation to alleviate disease initiation and progression, including but not limited to AS.^21^ A disruption of MMP directly affects the electron transport chain, resulting in oxidative stress, and its alteration triggers apoptosis and activates the NLRP3 inflammasome, aggravating AS.^22^ Particularly, Zhao L and his colleagues have elucidated the anti-inflammation property of Pue in coronary heart disease partially by reducing the production of TNF-α and IL-6.^23^ Meanwhile, in rats with chronic heart failure, Pue shows the ability to decrease TNF-α and IL-6.^24^ Notably, it has been determined that a crystal form of Pue, Pue-V can suppress inflammatory milieu in the myocardium of myocardial infarction mice, thereby limiting the upregulation of proinflammatory cytokines.^25^ Pue alleviates hypoxia-reperfusion-induced oxidative stress, reduces MDA content, enhances the antioxidant defense system, and increases SOD activity, and GSH levels.^26^ Pue preconditioning inhibits excessive oxidative stress and inflammatory cytokine release and maintains mitochondrial function to alleviate lipopolysaccharide-induced myocardial injury.^27^ Interestingly, Pue preconditioning can reduce doxorubicin-induced cardiotoxicity by inhibiting excessive oxidative stress and maintaining mitochondrial function.^28^

Analysis from a histopathological point of view found that mice exposed to acrolein developed atherosclerotic plaques in their aortas, accumulated lipids and developed steatosis in the livers, while Pue alleviated these symptoms. Consistently, Pue treatment combined with Tanshinone IIA can prevent atherosclerotic inflammation and delay AS pathology.^29^ Similarly, Pue improves liver lipid accumulation of nonalcoholic fatty liver disease rats^30^ and ameliorates hepatic steatosis by reducing lipid accumulation in hepatocytes.^31^

In terms of mechanism, this report determined the inhibition of Pue on the JNK signaling pathway to upregulate HDAC1 expression, thereby improving mitochondrial function. JNK pathway is activated in AS,^32^ and the inactivation of the JNK pathway confers an anti-atherosclerotic effect.^33^ It is believed that JNK partially regulates HDAC1/2-mediated inflammatory gene expression.^34^ However, this research did not further determine the action of JNK-mediated HDAC1 in AS, which needs further investigation.

In summary, the current research has delineated the protective mechanism of Que in AS by inactivating the JNK signaling pathway to alleviate HDAC1-induced oxidative stress disorder. These study findings have provided a novel reference basis for drug treatment in AS. Given that this study was based on animal studies, further validation of personalized clinical applications is needed.

## Availability of data and materials

The datasets used and/or analyzed during the present study are available from the corresponding author on reasonable request.

## Ethics approval

The present study was approved by the Animal experiments were approved by Dongying People's Hospital (Dongying Hospital of Shandong Provincial Hospital Group) and all procedures complied with the National Institutes of Health Guide for the Use of Laboratory Animals (nº 20200375DY).

## Authors' contributions

YeTing Li and XiaoNing Li conceived and designed the evaluation and drafted the manuscript. Man Zheng and ChunYan Xiang participated in designing the evaluation, performed parts of the statistical analysis and helped to draft the manuscript. FanLi Bu and FengLei Zhang re-evaluated the clinical data, revised the manuscript and performed the statistical analysis and revised the manuscript. YeTing Li and XiaoNing Li collected the clinical data, interpreted them and revised the manuscript. YeTing Li and XiaoNing Li re-analyzed the clinical and statistical data and revised the manuscript. All authors read and approved the final manuscript.

## Funding

Not applicable.

## Declaration of competing interest

The authors declare no conflicts of interest.
